# Protein ADP-Ribosylation Takes Control in Plant–Bacterium Interactions

**DOI:** 10.1371/journal.ppat.1005941

**Published:** 2016-12-01

**Authors:** Baomin Feng, Chenglong Liu, Libo Shan, Ping He

**Affiliations:** 1 Department of Biochemistry and Biophysics, and Institute for Plant Genomics and Biotechnology, Texas A&M University, College Station, Texas, United States of America; 2 Department of Plant Pathology and Microbiology, and Institute for Plant Genomics and Biotechnology, Texas A&M University, College Station, Texas, United States of America; The Sainsbury Laboratory, UNITED KINGDOM

## Introduction

Sessile plants detect and ward off invading microorganisms with a robust and sophisticated innate immune system in addition to structural, physical, and chemical barriers [1]. The first line of the plant immune system depends on pattern-recognition receptors (PRRs) that recognize conserved pathogen- or microbe-associated molecular patterns (PAMPs or MAMPs) and induce pattern-triggered immunity (PTI) [2]. To counteract this defense, pathogenic microbes have developed various virulence strategies, such as the bacterial type III secretion system (T3SS), through which bacteria inject a battery of effector proteins into plant cells to suppress host immunity and modulate host physiology [3]. Plants have, in turn, evolved intracellular NOD-like receptors (NLRs) that recognize effectors or effector-mediated changes and mount effector-triggered immunity (ETI) [1]. Recent studies show that protein ADP-ribosylation, an important yet less studied posttranslational modification with an emerging role in diverse cellular processes, is exploited both by plants to launch effective defense and by bacteria to achieve stealthy attacks to the hosts. Here, we summarize the classification and biochemical processes of protein ADP-ribosylation, compare the similarities and differences of ADP-ribosylation in plants and animals, and discuss the roles of ADP-ribosylation in plant immunity and bacterial pathogenicity.

## Protein ADP-Ribosylation: Biochemical Classification and Processes

**Fig 1:**
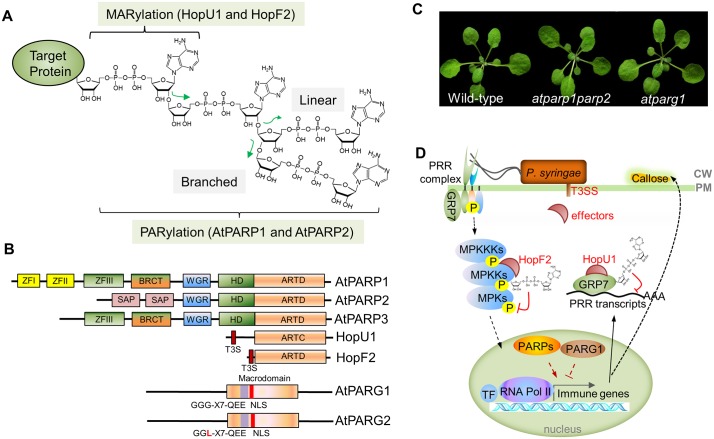
Protein ADP-ribosylation in plant–bacterium interactions. **(A)** Scheme of protein ADP-ribosylation. Covalent attachment of NAD^+^-derived ADP-ribose monomer or polymer to a target protein is MARylation or PARylation. The *Pseudomonas syringae* effectors HopU1 and HopF2 confer MARylation activity. *Arabidopsis* AtPARPs bear PARylation activity. Poly(ADP-ribose) chain could be linear or branched. **(B)** Domain architectures of AtPARPs, AtPARGs, and bacterial effectors HopU1 and HopF2. Abbreviations: ZF, zinc finger domain; WGR, tryptophan-glycine-arginine domain; BRCT, BRCA1 C-terminal domain; HD, helical subdomain; ART, (ADP-ribosyl)transferase domain; SAP, SAF-A/B, Acinus and PIAS domain; NLS, nuclear localization signal; T3S, Type III secretion signal. GGG-X7-QEE is PARG signature. **(C)** Growth phenotype of *Arabidopsis thaliana* wild-type Col-0, *atparp1parp2*, and *atparg1* mutants. Plants were grown at 23°C, with 45% humidity, 75 μE m^-2^s^-1^ light, and 12-hour light/12-hour dark photoperiods. The picture was taken at three weeks after germination. **(D)** A model of protein ADP-ribosylation in plant–bacterium interactions. Perception of MAMPs by PRR complexes activates immune gene transcriptional reprograming via activation of MAP kinase cascades (MPKKKs-MPKKs-MPKs) and leads to various defense responses including callose depositions at the cell wall. Reversible PARylation mediated by PARPs and PARG1 modulates *Arabidopsis* immune gene transcriptional reprograming. Bacterial effectors HopU1 and HopF2 MARylate GRP7 and MKK4/5, respectively, resulting in reduced PRR transcripts and proteins and MAP kinase inactivation to suppress plant immunity. CW, cell wall; PM, plasma membrane; T3SS, Type III secretion system; P, phosphorylation; TF, transcription factor.

ADP-ribosylation is the covalent attachment of ADP-ribose monomer (MAR) or polymer (PAR) derived from nicotinamide adenine dinucleotide (NAD^+^) to a target protein, which is termed mono(ADP-ribosyl)ation (MARylation) or poly(ADP-ribosyl)ation (PARylation), respectively (Fig 1A) [4]. MARylation and PARylation differ not only in the length of the ADP-ribose chain, but also in the enzymes catalyzing the reactions and subcellular localization of reactions [5]. MARylation is usually catalyzed by mono(ADP-ribosyl)transferases (ARTs), which were originally discovered as bacterial toxins, such as diphtheria toxin and *Pseudomonas aeruginosa* exotoxin A, and were classified into the H-Y-E, variant H-Y-E and R-S-E groups (H: histidine; Y: tyrosine; E: glutamate; R: arginine; S: serine) based on the conserved motifs in the catalytic domains [6]. In ART, the active-site H-Y-E motif is part of the binding pocket for NAD^+^. The invariant Glu (E) is a key catalytic residue that coordinates the transfer of ADP-ribose to the acceptor site, and His (H) facilitates the binding of NAD^+^. The Glu (E) in R-S-E type ART is also a key catalytic site that is aided by the Arg (R) and Ser (S) residues to position and stabilize NAD^+^-binding pocket [6,7]. ARTs in eukaryotes are classified as secreted or plasma membrane-anchored ectoenzymes (cholera toxin-like ADP-ribosyltransferases [ARTC]) and cytoplasm-localized intracellular enzymes (diphtheria toxin-like ADP-ribosyltransferases [ARTD]) [8]. PARylation is usually catalyzed by poly(ADP-ribosyl) polymerases (PARPs), which are much more prevalent in eukaryotes than in prokaryotes. PARPs catalyze both the initial MARylation and subsequent elongation of the ADP-ribose chain (PARylation), predominantly on glutamate (E), aspartate (D), arginine (R), or lysine (K) residues of an acceptor protein. Interestingly, among 17 human PARPs, most of them are shown or predicted to be able to catalyze the attachment of MAR to acceptor proteins, which are functional ARTs and were reclassified as ARTDs recently [9]. PARylation is a reversible process, and the covalently attached PAR could be removed by poly(ADP-ribose) glycohydrolases (PARGs), which contain both endo- and exo-glycohydrolase activities (Fig 1A), or by the relatively less-studied ADP-ribosyl hydrolase (ARH). The terminal ADP-ribose, or MAR, of acceptor proteins can be hydrolyzed by certain macrodomain proteins, such as MacroD1, MacroD2, and the terminal ADP-ribose protein glycohydrolase (TARG1), in humans [10]. ADP-ribose released from the hydrolysis of MAR or PAR could be further cleaved to adenosine monophosphate (AMP) and ribose-5-phosphate by nucleoside diphosphate-linked to some moiety-X (Nudix) hydrolases [11]. Protein PARylation regulates a wide range of cellular responses, including DNA damage detection and repair, chromatin remodeling, gene transcription, and protein localization and degradation [4]. Compared to PARylation, MARylation is less understood in eukaryotes, with emerging roles in the regulation of NF-κB signaling, gene transcription, and unfolded protein response [8].

## ADP-Ribosylation: Similarities and Differences in Plants and Animals

As in their mammalian counterparts, plant PARPs and PARGs are implicated in DNA repair, cell cycle, genotoxic stress, circadian rhythms, and gene regulation [11–13]. In contrast to the 17 PARPs in humans, the reference plant *Arabidopsis* genome encodes three PARPs (AtPARP1, AtPARP2, and AtPARP3) with the conserved ARTD motif (Fig 1B) [11,12]. AtPARP1 bears the highest homology to HsPARP-1, which is the most active, ubiquitous, and abundant member of PARPs in humans. However, *Arabidopsis* AtPARP2 is enzymatically more active than AtPARP1 in both in vitro and in vivo assays and plays a predominant role in DNA damage responses and plant immunity [14,15]. AtPARP3 is primarily expressed in seeds [15]. No putative ART has yet been found in plants. Interestingly, land plants possess a group of conserved and plant-specific PARP-like family proteins, with the PARP signature carrying the characteristic catalytic triad, H-Y-E, including Radical-induced Cell Death 1 (RCD1), Similar-to-RCD-One (SROs), and RCD-like proteins [16]. Although the *Arabidopsis* RCD1 was shown to be enzymatically inactive, the wheat SRO protein Ta-sro1 confers PARP activity in vivo and in vitro [17], suggesting the existence of additional and functional PARPs in plants. This may provide an explanation as to why the *Arabidopsis atparp1parp2* mutant is phenotypically indistinguishable from the wild-type plants at the vegetative growth stage (Fig 1C), whereas the mouse *parp1parp2* mutant is embryonically lethal. Compared to only one *PARG* gene in humans and mice, *Arabidopsis* contains two *PARGs* (*AtPARG1* and *AtPARG2*) (Fig 1B). AtPARG1 shows strong PAR glycohydrolase activity, and AtPARG2, which carries a polymorphism in the PARG signature motif, is likely enzymatically inactive [15,18]. The ARH hydrolase has not been found in plants. In addition, there are no plant homologs of MacroD or TARG1, which removes the terminal ADP-ribose or MAR of acceptor proteins. Furthermore, in contrast to the embryonic lethality of the mouse *parg* mutant, the *Arabidopsis atparg1* mutant phenotypically resembles WT plants in terms of growth and development (Fig 1C). It is possible that additional enzymes with PAR or MAR hydrolase activity yet, without conventional PARG motifs, exist in plants.

## Poly(ADP-Ribosyl)ation: An Emerging Player in Plant Immunity

Recent studies suggest the essential role of protein PARylation in plant immunity [14,15,19,20]. A transcriptomic analysis of plant ETI responses identified *AtPARG2* as one of the highly induced genes in multiple plant–bacterium interactions [20]. Interestingly, the *atparg1* mutant, but not the *atparg2* mutant, showed augmented immune responses, including enhanced lignification and seedling growth inhibition, upon MAMP elicitation [15,19], in line with the observation that AtPARG1, but not AtPARG2, is enzymatically active. A forward genetic screen using an early PTI marker gene as a reporter uncovers an essential role of PARylation in plant PTI-mediated immune gene expression. The *atparg1* mutant displayed elevated early and late PTI responses, including flagellin-induced immune gene expression, lignification, and callose deposition [15]. In addition, the *atparp1parp2* double mutant showed reduced flagellin-induced immune gene expression and increased susceptibility to bacterium *Pseudomonas syringae* infections [14,15]. Importantly, the enzymatic activity of AtPARP2 could be stimulated upon MAMP treatment, reinforcing its role in PTI signaling [15]. It was reported that pathogen infections trigger host DNA double-strand breaks (DSBs) [21], which might potentially serve as a trigger to activate PARP. However, such DSBs were not detected upon PAMP perception, suggesting that PARP activation in PTI is modulated by other mechanisms. The study with PARP inhibitors also suggests a positive role of protein PARylation in multiple PTI responses, including flagellin-induced callose deposition [19]. The effect of protein PARylation on callose deposition might be due to the transcriptional regulation of callose biosynthetic genes [22]. The Nudix hydrolase NUDX (also named NUDT) family proteins also play an important role in fine-tuning plant immune responses [11]. The AtNUDX7 negatively regulates plant PTI and ETI [20,23,24]. The over-activation of defense in the *atnudx6nudx7* mutant might be due to up-regulation of an NLR gene *SNC1* and imbalance of ammonium/nitrate ratio in growth medium [25]. Interestingly, AtNUDX6 and AtNUDX7 have distinct roles in stress responses and accumulation of plant defense hormone salicylic acid (SA) and its induced genes [26]. Further studies might reveal the mechanistic connections among Nudix hydrolases, free PAR pool, and plant immune responses.

## Mono(ADP-Ribosyl)ation: A Weapon for Bacterial Pathogenicity

Bacterial T3SS effectors have been discovered as diverse biochemical weapons to dampen plant immune signaling via modifying host proteins [3]. Two *P*. *syringae* effectors HopU1 and HopF2, both of which are important virulence factors, carry the ART activity towards different targets (Fig 1D) [27,28]. The crystal structure of HopU1 reveals that it possesses R-S-E type (ARTC) catalytic core and shares structural similarity to the C3 type bacterial ART toxins *Clostridium limosum* C3 exoenzyme and *Clostridium botulinum* ART C3bot2 [6,29]. Notably, HopU1 carries two unique protruding loops, which are essential for substrate binding and enzymatic activity [29]. HopU1 MARylates multiple RNA-binding proteins, some of which, such as glycine-rich RNA-binding protein GRP7, are important in plant immunity [28]. GRP7 associates with multiple PRR proteins and transcripts [30]. MARylation of GRP7 at the conserved arginine 49 (R49), which is located within the RNA binding domain, by HopU1 is able to disrupt its binding to PRR transcripts, not to PRR proteins, and results in the reduced PRR protein accumulation upon bacterial infections [30].

HopF2 is an H-Y-E type ART (ARTD), structurally similar to diphtheria toxin and MARylates multiple MAP kinase kinases (MKKs), some of which, such as MKK4 and MKK5, are key signal transducers in relaying PTI signaling (Fig 1D) [27]. MARylation of MKK5, likely at arginine 313 (R313), inhibits its kinase activity towards downstream MAP kinases, thereby suppressing multiple MAMP-induced responses. Mutations of the putative ART motif affect both virulent and avirulent functions of HopF2 and its homolog HopF1 in different *P*. *syringae* bacteria [27]. In addition, *P*. *syringae* effector AvrRpm1 has a loosely defined H-Y-E motif and has been hypothesized to function as an ART [31]. It appears that MARylation of host proteins by effectors is an effective way for bacteria to interfere with plant immune signaling and facilitate infections.

## Concluding Remarks

Protein ADP-ribosylation as a versatile molecular switch has been utilized by plants as an intrinsic regulatory mechanism for immunity and by bacteria as an effective invasion strategy for pathogenicity. Meanwhile, many outstanding questions underlying these processes still remain to be addressed. Are there any additional ARTs and hydrolases in plants? What are the enzymes that remove the terminal ADP-ribose of target proteins in plants? How are PARP and PARG activities regulated in plant immunity? Human HsPARP-1 is regulated by multiple posttranslational modification processes, such as phosphorylation, ubiquitination, SUMOylation, and cleavage [32]. It remains unknown whether a similar activation mechanism exists for plant PARPs. What are the targets of protein PARylation and what are their functions in plant immunity? Are there any other effectors from bacteria or other pathogens possessing the ART or PARP activity? Elucidating these questions will reveal the detailed mechanisms underlying protein PARylation and MARylation in plant–pathogen interactions.
